# The Old and the New

**Published:** 1914-05

**Authors:** Walter G. Doty


### The Old and the New.

BY WALTER G. DOTY.

She fell in love with an aviator,
His dashing ways quite turned her head;
Her father swore he'd never mate her,
"He's far too flighty," the old man said.

But the girl replied: "In his own profession
He's rising rapidly every day;
His outlook's broad and his standard lofty,
And he has the power to make his way.

"He's fitted to move in the highest circles—
Count not my love but a passing whim;
When my daring hero gives me the signal,
I'm ready and eager to fly with him."

The two eloped in a biplane airy,
But the thing broke down in a little while.
Then into an auto that stood there handy,
But a tire blew up ere they'd gone a mile.

In a motor boat they attempted crossing
A stream that ran through a valley fair;
But the engine balked in a vicious manner,
And the father caught up with the old bay mare.

—*Farm Journal.*

